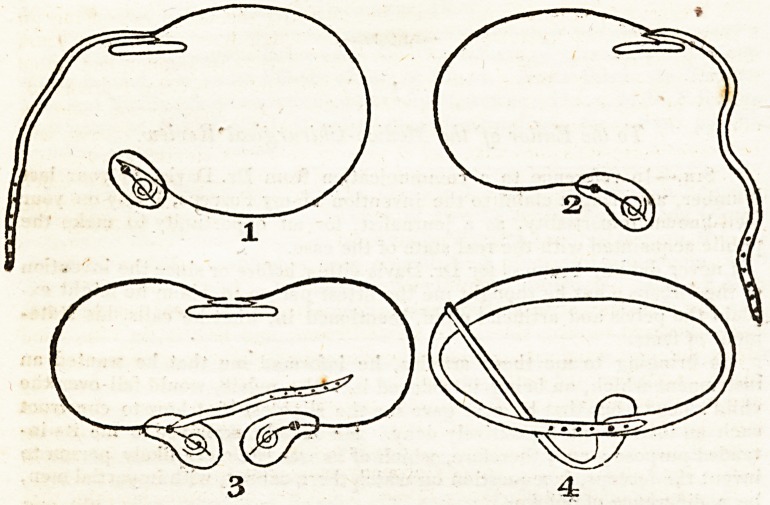# Extra-Limites

**Published:** 1826-01

**Authors:** 


### EXTRA-LIMITES.

### I.

*To the Editor of the Medico-Chirurgical Review.*

Sir.—In reference to a communication from Dr. Davis, in your last Number, asserting a claim to the invention of my Forceps, I rely on your well-known impartiality, as a journalist, for an opportunity to make the public acquainted with the real state of the case.

I never did any business for Dr. Davis either before or since the invention of the forceps; but he thought me the fittest person to whom he might explain the pelvis and artificial child, mentioned in, what he calls, his statement of facts.

On bringing to me these articles, he informed me that he wanted an instrument which, on being introduced into the pelvis, would fall over the child's head; but that he ever gave me the slightest hint how to construct such an instrument I positively deny. He merely explained to me its intended purpose; and, therefore, which of us was the more likely person to invent the forceps, is a question on which there cannot, with impartial men, be a difference of opinion.

Among other things, Dr. Davis has taken occasion to say, that the vectis was made to the order of a gentleman from the country; but he must be well aware that this gentleman never pretended to claim the merit of the invention. It would be strange, indeed, if such a claim were allowed, and no less strange if a person, coming to my house to say that he wanted an instrument adapted to fall over a child's head, and imposing on me the trouble and expense of bringing it to perfection, should ascribe to himself the whole credit of it, merely because he had conceived the wonderful notion that it would be useful.

I can, with truth, affirm that, before I perfected the instrument, I made five or six different attempts, at an expense of more than twenty Pounds in workmen's wages alone; yet I have never seen the colour of the Doctor's money. I should have had no objection to have made him a present of the invention, if he had conducted himself towards me in that handsome manner which I had reason to expect in a gentleman; but, on the contrary, he explored the whole town to procure a man that works for the trade, that he might get the instrument made a little cheaper. What kind of encouragement this is to persons who freely expend their time and money for the public good, I leave the public to decide.

I am, Sir,

Your most obedient Servant,

JOHN WEISS.

### II.

### Mr. COLES' TRUSSES.

*Explanation of the Drawings.*

Fig. 1. Is an opposite-sided Truss, the Spring having a returned end in front, to which the pad is fixed, with two screws, to the spiral spring *in the pad,* which effects a resistance to the descent of the hernia; a cushion presses in the centre of the back.

A Spring Truss for the navel differs from the above only by having a circular pad, with two spiral rings, in front, and the end of the outer spring bent down square.

A Pad of the latter description is more generally worn to a bandage, in navel ruptures, the Springs in the Pad possessing sufficient pressure, without any external spring or back-cushion.

Fig. 2. Is a Half-truss: the Cushion presses on the centre of the back; the front pad effects a resistance as above described. Its construction is more simple than the former, and generally worn in cases of femoral or inguinal hernia; a strap forms the other half of the above Trusses, and is looped on to the pad in front.

Fig. 3. Is a double Truss, united to a Cushion in the back, capable of being diminished or increased in size, and, like the former, is proper to be worn in cases of femoral or inguinal hernia; neither case requires any under strap.

Fig. 4. Is a Bandage, having no external spring, and only one spiral spring in the centre of the pad. The girdle has a piece of steel stitched into one end, which is screwed to the spiral spring; the other end passes through the thigh strap to the hip bone opposite to the complaint, and is looped on to the front pad, by which means its pressure is that of a lever acting upon the spiral spring: the pad is formed to fit the os pubis, and is intended not only to press upon the opening above the pubis, but to press upon and close the aperture through which many an old hernia will escape in defiance of all the self-adjusting or self-resisting qualities of either of the above, or any other description of Trusses; in proof of which, he begs to submit the following testimonials, beginning with Mr. Earle's certificate.

"I hereby certify, that Mr. Coles has perfectly succeeded in keeping up a very difficult and complicated Rupture, on which I operated some years since, in which case, many of the best Truss Makers in London had failed to afford any relief. When I placed this Patient under his care, I promised, should he succeed, to give him a Certificate of my approbation, and, in conformity with that, I now state my firm conviction that his Trusses will be found more efficacious than any at present employed in similar cases. I have since tried his Trusses in more recent Cases, and have been much pleased with the ingenuity he has displayed in adapting his means to the circumstances of each case. H. EARLE.

"George-street, Hanover-square."

*To Mr. W. C. Coles.*

Sir.—A lady of this town having consulted us for her son, who has been ruptured on one side since last October; and having, in vain, tried three different elastic steel trusses, besides others without springs, to keep his rupture from descending, found the one you sent us most satisfactorily and most comfortably to succeed, the rupture not having come down once since we first applied it in March last. One of the greatest advantages in your Trusses is, that people can conveniently sleep in them, by which means they obtain a cure, though of many years' standing.

P.S. I recollect, in Sir Astley Cooper's lectures on hernia, he said, that if constant pressure could be made on the herniæ by night and by day, he had no doubt but very old herniæ might be radically cured by it.

Dartmouth, Your most obedient servants,
July 17th, 1824. W. C. HUNT & SON.

Mr. Hillman feels pleasure in recommending Mr. Coles's self-adjusting Truss, as being, in his opinion, the best instrument in use at this time, from its affording an uniform pressure under all the positions of the body.

Argyll-street, 8th July, 1824.

I hereby certify that I have worn the Trusses of Mr. Coles's invention for 16 months past, and have found them more easy and more efficacious than any others which I had worn for 14 years previously.

London, Sept. 20th, 1824. CARYER VICKERY, Surgeon, R.N.

I hereby certify that, in various cases of Hernia, I have tried Mr. Coles's Trusses, and I find them very superior to any I ever used.

Newington, Aug. 30th, 1824 M. L. MASON.
Member of the Royal College of Surgeons.

---

The following Affidavits have been recently sworn at the Mansion House.—

*London to Wit.*—George Carpenter, of Bath-row, near Birmingham, gentleman, maketh oath and saith, that he has been afflicted with a Rupture forty years, during which period he has consulted many eminent men of the faculty, amongst whom are Mr. Hodson, Mr. Barr, and Dr. De Lis, of Birmingham, Mr. Estlin, of Bristol, and Henry Earle, Esq. of St. Bartholomew's Hospital, London; and that he has used a variety of different kinds of trusses, both patent and common, all of which entirely failed to relieve him of his infirmity; and one of the abovenamed gentlemen gave his decided opinion that no instrument on earth was calculated to arrest its progress; and this deponent despaired ever obtaining any relief. Although deponent is but small in stature, the rupture exceeded 18 inches in circumference, and he had made no effort to reduce it for several years past. It has been conjectured that the bladder formed a part of the tumour, and, from its contracted state, rendered an evacuation indispensible almost every hour throughout the night, which has deprived him of enjoying natural rest for several years past, and caused the most exquisite torture, at times, both in body and mind, from which he had no conception that any thing but death could release him. Happily, however, a circumstance occurred which brought him to London; and, immediately on his arrival, he was advised, by a gentleman to whom he was a perfect stranger, to try Coles's Patent Truss of London Bridge; and this deponent has now the satisfaction of being able to convince the world that Mr. Coles has effectually succeeded in delivering him, now in his 78th year, from the least appearance of this dreadful affliction: and this deponent further maketh oath, that he would not part with the instrument with which Mr. Coles has furnished him, and remain in his former state, if any person would give him its weight in gold.

(Signed) GEORGE CARPENTER.

*Sworn at the Mansion House, this 13th day of July, 1825, before me, John Garratt, Mayor.*

*London to Wit.*—Nicholas Boylston, maketh oath and saith, that he has been afflicted with a very painful rupture upwards of twenty years, during which period he had made use of almost every kind of truss, but from their incapacity to secure one rupture the whole system became deranged, and a second rupture took place which rendered him completely miserable, and utterly incompetent to fulfil the duties of his employ, until, by mere chance, he saw a written inscription containing the opinion of Dr. Birkbeck in reference to Coles's Patent Bandages and Trusses, and he instantly resolved to make trial of it; and this deponent further maketh oath, that the bandage with which Mr. Coles has furnished him has performed its office well ever since he first applied it in April last, and has not produced a painful sensation, which fully demonstrates that bad ruptures, when properly treated, can scarcely be called afflictions; and this deponent is willing that the world may know he is ruptured, and he will satisfy any person who may be desirous to ascertain the fact of his being so ruptured whenever they please. (Signed) NICHOLAS BOYLSTON.

*Sworn at the Mansion House, this 8th day of August, 1825, before me, John Garratt, Mayor.*

*London to Wit*—Richard Johnson, of the parish of Stoke upon Trent, wholesale dealer in Staffordshire Ware, maketh oath and saith, that he has been afflicted with a very painful rupture, which has baffled every truss and bandage that he has employed for twenty-five years past. Deponent further saith, that, notwithstanding his affliction he has continued travelling for orders through a great part of the kingdom, and upon various accidents arising from the above complaint, he has been compelled to take the advice of the most eminent of the faculty in London and different parts of the kingdom; and with all their advice he never yet found a man who possessed skill enough to construct an instrument which was capable of preventing its enlargement, until Mr. Coles, of London Bridge, furnished him with one of his invention; and this deponent further maketh oath, that the bandage he now wears is of more value in his case than all the trusses he had previously used.

RICHARD JOHNSON,
16, Butler's Buildings, Horsleydown, London.

*Sworn at the Mansion House, this 30th day of September, 1825, before me, John Garratt, Mayor.*

*London to Wit.*—Robert Wilson, of Dartmouth-street, in the parish of St. Margaret, Westminster, Gentleman, aged fifty-five, maketh oath and saith, that he has been afflicted with a violent rupture upwards of forty years, and that during this long period, he has repeatedly applied to several eminent surgeons and others without obtaining the least relief. Deponent also saith, that about eight years ago he applied to the late Mr. Taunton, of Hatton-garden, surgeon to the City of London Truss Society, who furnished him with a common truss, stating that it was the only thing that could afford relief; but deponent after wearing it some time was obliged to abandon it, as he was not only without any benefit, but got much worse by the use of it. In this state deponent applied to Mr. Coles, at the foot of London Bridge, who furnished one of his bandages, which deponent has now worn for several months past with a degree of comfort and convenience before unknown; and he is in every respect much better than he could have expected.

ROBERT WILSON.

*Sworn at the Mansion House, the 14th day of November,* 1825, *before me W. Venables, Mayor.*

N.B. Private door in Thames-street, corner of London Bridge.

*** In all cases, whether navel, femoral, inguinal, or scrotal Hernia, the particulars of the complaint should be stated, and whether the patients are stout or thin in their persons—whether they have single or double ruptures, and which side they are afflicted—if both sides, which side is worst.

## Figures and Tables

**1 2 3 4 f1:**